# Efficient generation of brain organoids using magnetized gold nanoparticles

**DOI:** 10.1038/s41598-023-48655-8

**Published:** 2023-12-01

**Authors:** Hongwon Kim, Yoo-Jung Lee, Youngeun Kwon, Jongpil Kim

**Affiliations:** 1 Laboratory of Stem Cells & Gene Editing, Department of Chemistry, Dongguk University, Pildong-Ro 1-Gil 30, Jung-Gu, Seoul, 04620 Republic of Korea; 2 Department of Chemistry and Chemical Biology, Rutgers, The State University of New Jersey, Piscataway, NJ 08854 USA; 3 Laboratory of Protein Engineering, Department of Biomedical Engineering, Dongguk University, Seoul, 04620 Republic of Korea

**Keywords:** Reprogramming, Biomaterials

## Abstract

Brain organoids, which are three-dimensional cell culture models, have the ability to mimic certain structural and functional aspects of the human brain. However, creating these organoids can be a complicated and difficult process due to various technological hurdles. This study presents a method for effectively generating cerebral organoids from human induced pluripotent stem cells (hiPSCs) using electromagnetic gold nanoparticles (AuNPs). By exposing mature cerebral organoids to magnetized AuNPs, we were able to cultivate them in less than 3 weeks. The initial differentiation and neural induction of the neurosphere occurred within the first week, followed by maturation, including regional patterning and the formation of complex networks, during the subsequent 2 weeks under the influence of magnetized AuNPs. Furthermore, we observed a significant enhancement in neurogenic maturation in the brain organoids, as evidenced by increased histone acetylation in the presence of electromagnetic AuNPs. Consequently, electromagnetic AuNPs offer a promising in vitro system for efficiently generating more advanced human brain organoids that closely resemble the complexity of the human brain.

## Introduction

Brain organoids offer a remarkable opportunity to closely mimic the complexity of the human brain and study its development, model diseases, and screen potential drugs^1,2^. These organoids are typically derived from pluripotent stem cells that are guided to differentiate into various types of brain cells, including neurons and glial cells. Through self-organization, these cells form intricate networks that resemble the developing human brain^3,4^. Consequently, brain organoids have emerged as a valuable tool for investigating brain development, disease mechanisms, and drug responses. For instance, researchers have developed cerebral and midbrain organoids to overcome the challenges associated with limited access to and complexity of human brain tissues^5–7^. Furthermore, the application of neurological disease organoid models, such as those for Alzheimer's, Parkinson's, Autism, and Huntington's disease, has significantly contributed to our understanding of these conditions within a three-dimensional environment that closely resembles the human brain^8–15^. Thus, brain organoids hold immense promise for comprehending brain development, diseases, and serve as a valuable platform for disease modeling, drug screening, and translational medicine.

However, generating mature brain organoids is a time-consuming process involving multiple steps, including three-dimensional differentiation and maturation. The efficiency of producing fully developed brain organoids is significantly low, with many organoids failing to reach complete maturation or failing to develop altogether. Recent pioneering efforts have aimed to address these limitations and enhance the efficiency and reproducibility of brain organoid generation^16–18^. Notably, the utilization of the air–liquid interface system in the organoid culture has demonstrated improvements in neuronal survival and axon outgrowth within cerebral organoids^19^. Additionally, the development of brain organoid-on-a-chip technology has provided perfusable microfluidic platforms with multicellular architectures, facilitating the organization and neural differentiation of mature brain organoids^20^. Despite these significant advancements, generating brain organoids that faithfully replicate the characteristics of the human brain remains an ongoing challenge. The time-intensive nature of the process and the need for maturation present major obstacles to the broader application of brain organoids in research and clinical settings.

Previously, our studies have demonstrated the remarkable potential of extremely low-frequency electromagnetic fields (EL-EMF) or magnetized gold nanoparticles in neuronal fate conversion and direct neuronal reprogramming, significantly enhancing the efficiency of these processes^21,22^. In these studies, we observed a robust activation of the histone acetyltransferase, Brd2, resulting in the acetylation of histones H4K12 and H3K27 at the promoter regions of neuronal-specific genes. This activation ultimately improved the efficiency of neuronal reprogramming^21^. Moreover, another study involving electromagnetized AuNP stimulation in the hippocampus of EMF-exposed mice demonstrated increased histone acetylation, leading to the activation of adult neural stem cells and enhanced neurogenesis in an aged mouse model^22^. These findings highlight the potential of magnetized AuNPs as a promising strategy for improving neuronal cell fate determination and its implications in regenerative medicine.

**Figure 1:**
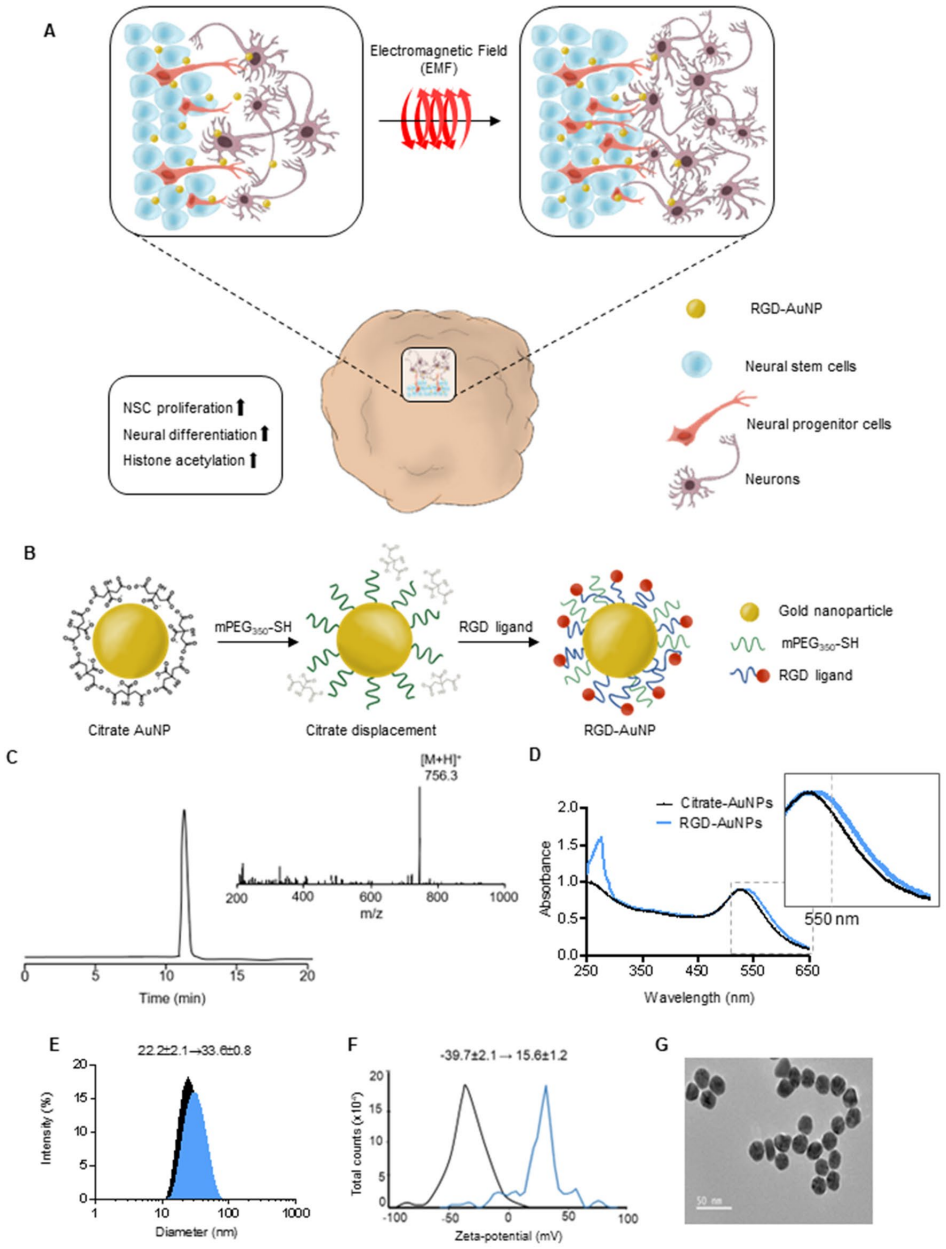
(**A**) Schematic presentation of the efficient generation of the brain organoids using electromagnetized AuNPs. The conjugation of RGD-AuNPs in brain organoids coupled with exposure to electromagnetized field can facilitate the proliferation and differentiation of neural stem cells and activate epigenetic histone modification, including H3K9ac. (**B**) Schematic depicting the main steps for magnetizable RGD (arginine-glycine-aspartic acid)-conjugated AuNPs synthesis. (**C**) Data showing HPLC chromatogram and MS (ion masses) of the synthetic peptide, CYGRGDS. Analysis of RGD-AuNPs by (**D**) UV–vis spectroscopy, (**E**) DLS, (**F**) Zeta-potential, and (**G**) FE-TEM analysis.

In this study, we present a highly efficient approach for generating mature 3D brain organoids by employing magnetized AuNPs. Our findings reveal a remarkable maturation of brain organoids within a mere 3-week period following treatment with magnetized AuNPs. Notably, these organoids closely resemble brain organoids cultured for 12 weeks using conventional methods. Our results demonstrate that magnetized AuNPs significantly enhance the efficiency and reproducibility of brain organoid generation, primarily through the upregulation of histone acetylation (Fig. 1A). Consequently, our study highlights the exceptional advantages of electromagnetic stimulation using AuNPs exposed to EL-EMF, as it offers scalability, reproducibility, and superior efficiency in generating organoids that closely emulate the intricate structure and function of the human brain.

## Results and discussion

### Preparation of magnetized AuNPs for inducing brain organoids

Cerebral organoids, representing a complex 3D cell culture model that partially recapitulates the characteristics of the human cerebral brain, hold immense potential for various applications^18^. To improve the generation efficiency of cerebral organoids using electromagnetized AuNPs, we employed a two-step preparation process to create magnetizable RGD (arginine-glycine-aspartic acid)-conjugated AuNPs (Fig. 1B). We chose to coat the AuNPs with RGD, which is a well-known binding motif found in fibronectins. This is driven by the fact that AuNPs have a limited surface area, necessitating surface modification to achieve a high density of tightly binding ligands, such as RGD^23^. This modification is crucial to enable efficient and specific coating for neuronal applications^24–26^. Following the reports from previous studies, we modified the AuNPs by incorporating thiol-containing ligands, mercapto (methoxypolyethylene glycol and mPEF350-SH), and RGD peptides. This modification aimed to induce magnetic polarization and enhance cell adhesion to the AuNP substrates (Fig. 1C)^27^. To assess the properties of RGD-conjugated AuNPs, we employed UV–Vis spectroscopy in conjunction with dynamic light scattering and ζ-potential measurements. The absorption spectrum of the RGD-conjugated AuNPs exhibited a shift towards 532 nm compared to the citrate-conjugated AuNPs (Fig. 1D). Additionally, the hydrodynamic radii of the AuNPs increased from 22.2 ± 2.1 nm to 33.6 ± 0.8 nm, and the ζ-potential shifted from − 39.7 ± 2.1 mV to 15.6 ± 1.2 mV upon the introduction of RGD-containing peptides (Fig. 1E,F). Field emission scanning electron microscopy (FE-SEM) analysis confirmed the well-dispersed distribution of the RGD-AuNPs (Fig. 1G).

**Figure 2:**
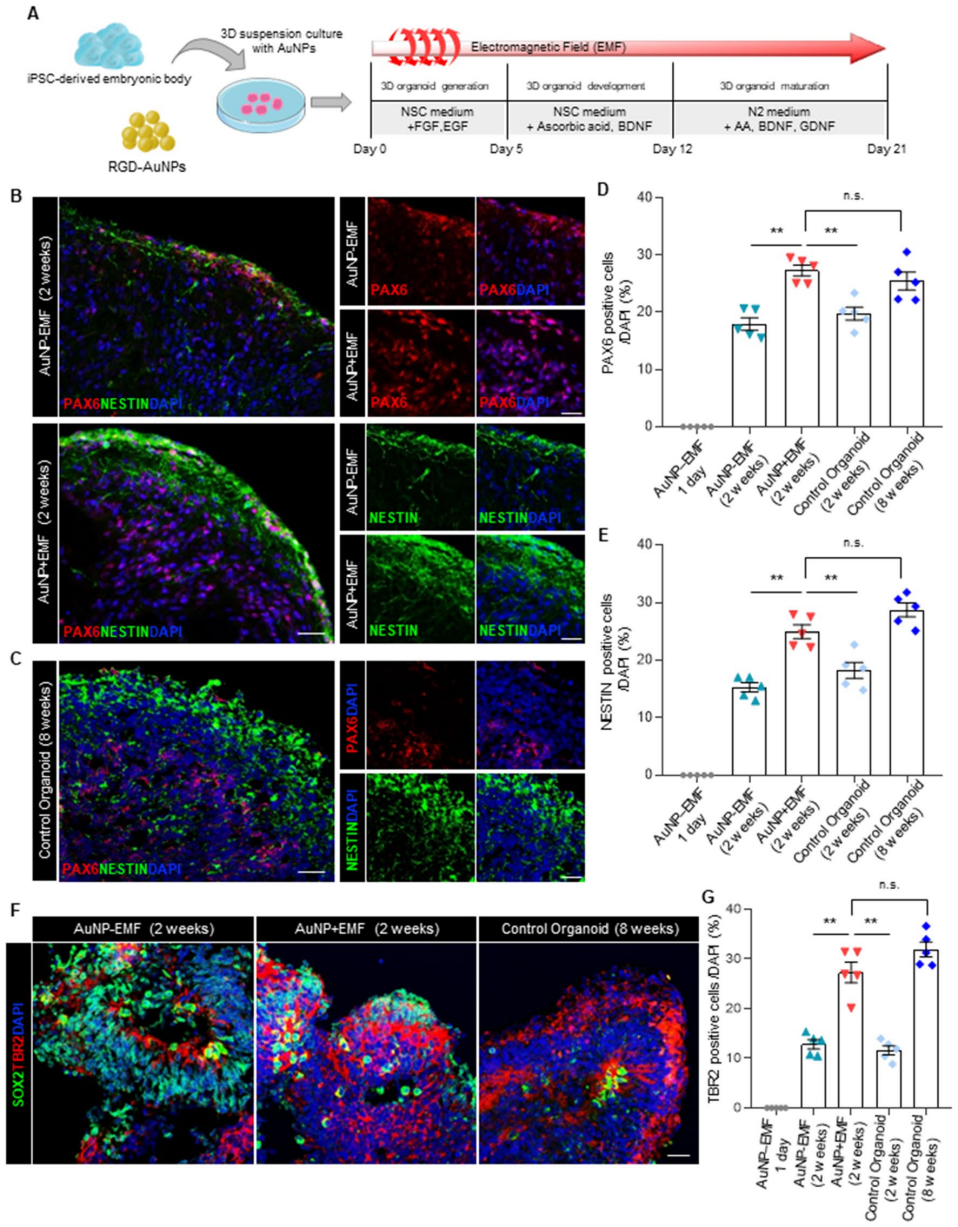
(**A**) Schematic depicting the main steps for electromagnetized AuNP-brain organoids production. To generate AuNP-brain organoids, iPSC-derived embryonic bodies, and RGD–AuNPs were gently mixed and then embedded in a Matrigel matrix for the structural organization. The brain organoids were exposed to an electromagnetized field (60 Hz and 1 mT EMF) for 6 h per day for 3 weeks. (**B**) Immunofluorescence for PAX6 and NESTIN in the control and electromagnetized AuNP organoids at 2 weeks. Scale bar = 50 µm. (**C**) Immunofluorescence for PAX6 and NESTIN in control organoids at 8 weeks. Scale bar = 50 µm. (**D**,**E**) Quantifications of the PAX6 + (**D**) and NESTIN + (**E**) cells in control organoids and electromagnetized AuNP organoids. Data represent mean ± SEM. one-way ANOVA, *P < 0.05, **P < 0.01; *n* = 5 per group. (**F**) Immunofluorescence for SOX2 and TBR2 in control and electromagnetized AuNP organoids. Scale bar = 20 µm. (**G**) Quantifications of the TBR2 + cells in control organoids and electromagnetized AuNP organoids. Data represent mean ± SEM. one-way ANOVA, **P < 0.01; *n* = 5 per group. *Control-EMF* brain organoid without AuNPs and EMF exposure, *Control* + *EMF* EMF-exposed brain organoid without AuNPs, *AuNP-EMF* AuNP-brain organoid without EMF exposure, *AuNP* + *EMF* AuNP-brain organoid with EMF exposure.

To investigate the potential of magnetized AuNPs in promoting the generation of cerebral organoids, we developed a modified protocol based on the self-organizing principles of human induced pluripotent stem cell (iPSC) cultures from previous studies^1,13^. Initially, a single iPSC-derived embryonic body was mixed with RDG-conjugated AuNPs at a concentration of 8 × 10^12^ nanoparticles/mL in Matrigel, forming a three-dimensional structure (Fig. 2A). Based on previous research indicating the neurogenic potential of EL-EMF exposures with a frequency of 60 Hz and an intensity of 1 mT, the 3D aggregated cultures were treated with epidermal growth factor (EGF) and fibroblast growth factor-8 (FGF8) under the same EMF condition for 5 days to promote the patterning of neuroectodermal fate. Subsequently, to induce the maturation of the cerebral organoids, the AuNP-treated organoids were exposed to the same EMF condition for additional 2 weeks in the presence of brain-derived neurotrophic factor (BDNF), glial cell-derived neurotrophic factor (GDNF), and ascorbic acid (Fig. 2A).

Remarkably, our observations revealed a significant increase in PAX6 + NESTIN + neural stem cells within the electromagnetized AuNP-treated group, indicating a significant enhancement of neurogenesis in the cerebral organoids after 2 weeks of exposure to EMF-exposed AuNPs (Fig. 2B–G). However, we did observe a slight increase in size compared to the EMF or AuNPs-only control group (Fig. S1A). Considering a previous study by Fayol et al. that reported EMF exposure-induced cell condensation and volume reduction during chondrogenic differentiation of stem cells^28^, we further confirmed cell proliferation within the electromagnetized AuNP brain organoids. Significantly increased Ki67-positive cells in the magnetized AuNP brain organoids suggest that EMF stimulation contributes to the augmented size of the AuNP organoids primarily through the activation of neuronal proliferation (Fig. S1B, C). Moreover, a cell viability assay was conducted to evaluate the cytotoxicity of AuNPs in the cerebral organoids, revealing no significant differences between the control group and the magnetized AuNPs-treated organoids (Fig. S1D).

### Promotion of cerebral organoid maturation by electromagnetized AuNPs

**Figure 3:**
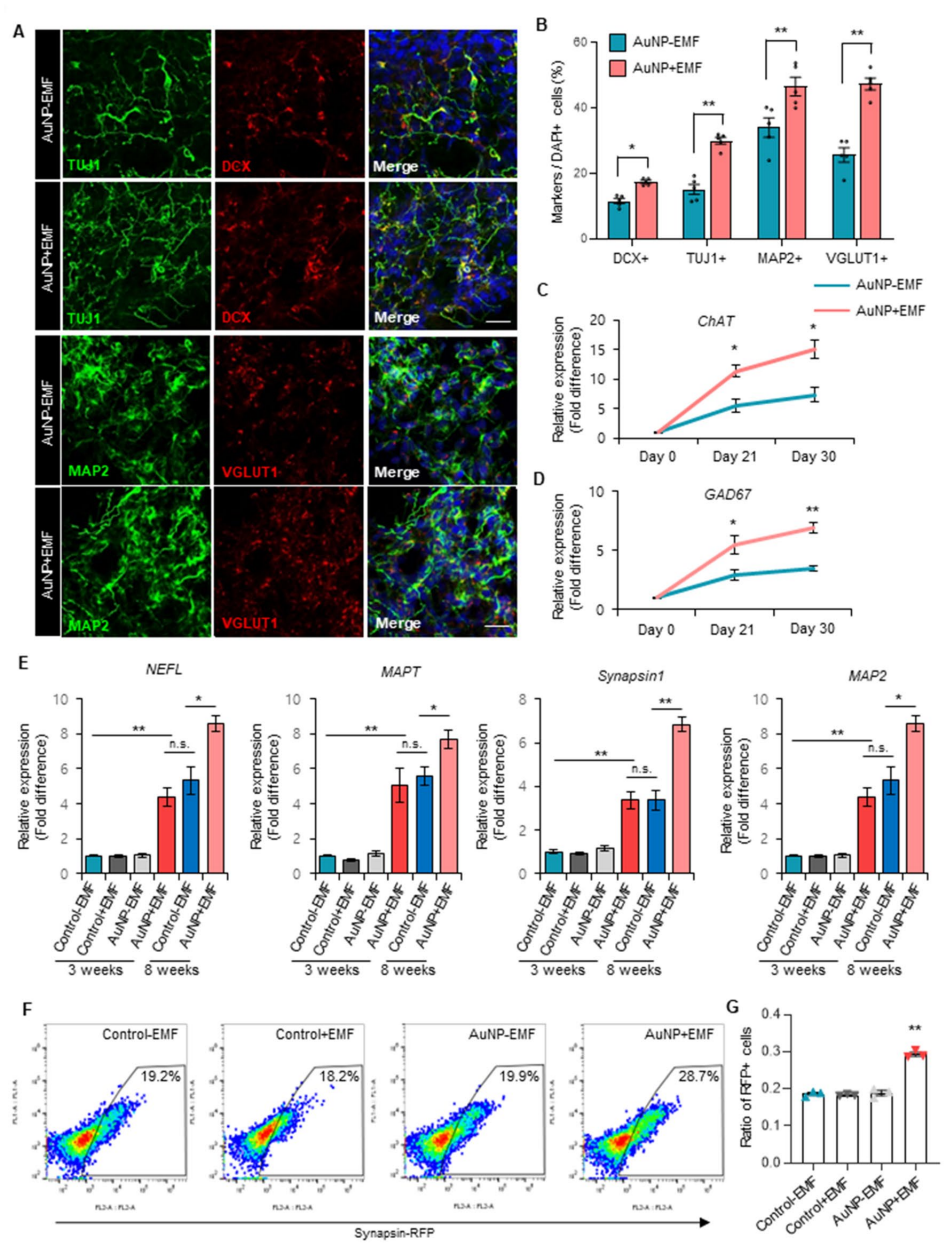
(**A**) Immunofluorescence for DCX+, TUJ1+, MAP2+, and VGLUT1 + neurons in brain organoids with or without electromagnetized AuNPs. Scale bar = 20 µm. (**B**) Quantification of DCX+, TUJ1+, MAP2+, and VGLUT1+ neurons in each condition at 3 weeks. Data represent mean ± SEM. Student’s t-test, *P < 0.05, **P < 0.01; *n* = 5 per group. (**C**,**D**) qRT-PCR analysis of the specific markers for cholinergic neurons, *ChAT* (C), and GABAergic neurons, *GAD67* (D), at different time points. Data represent mean ± SEM. Student’s t-test, *P < 0.05, **P < 0.01; *n* = 3 per group. (**E**) qRT-PCR analysis of neuronal markers including *NEFL*, *MAPT*, *Synapsin1*, and *MAP2* in control-EMF, control + EMF, AuNP-EMF, and AuNP + EMF organoids. Data represent mean ± SEM. one-way ANOVA, *P < 0.05, **P < 0.01; *n* = 3 per group. (**F**) Representative FACS plots of synapsin-RFP-positive cells from control-EMF, control + EMF, AuNP-EMF, and AuNP + EMF organoids. (**G**) Quantification of synapsin-RFP-positive cells in each group. Data represent mean ± SEM. one-way ANOVA, **P < 0.01; *n* = 3 per group. *Control-EMF* brain organoid without AuNPs and EMF exposure, *Control* + *EMF* EMF-exposed brain organoid without AuNPs, *AuNP-EMF* AuNP-brain organoid without EMF exposure, *AuNP* + *EMF* AuNP-brain organoid with EMF exposure.

To assess the impact of electromagnetized AuNPs on the functional maturation of cerebral organoids, we proceeded to examine the expression of marker genes associated with the maturation process. Initially, we observed a significant increase in the population of DCX+, TUJ1+, and MAP2+ neuronal cells in the brain organoids exposed to EMF for 2 weeks (Fig. 3A,B). Moreover, EMF-exposed AuNP organoids exhibited a substantial rise in VGLUT1+ mature glutamatergic neurons and CTIP2+ cortical-layer neurons (Fig. 3A,B, Fig. S1E). Consistently, RT-PCR analysis of markers for dorsal pallial (CTIP2 and SATB2), cholinergic (ChAT), and GABAergic (GAD67) neurons demonstrated significant increases in the brain organoids subjected to EMF, indicating the effective maturation of the organoids facilitated by magnetized AuNPs (Fig. 3C,D, Fig. S1F). Furthermore, mature neuronal markers including NEFL, MAPT, Synapsin1, and MAP2 exhibited significant upregulation after 3 weeks of EMF treatment in the cerebral organoids (Fig. 3E). Notably, the expression levels of these mature marker genes in brain organoids treated with magnetized AuNPs for 2 weeks closely resembled those in the control brain organoids cultured for 8 weeks using conventional methods (Fig. 3E). Moreover, the expression levels of these mature marker genes were significantly increased in brain organoids treated with magnetized AuNPs for 7 weeks compared to the control brain organoids cultured for 8 weeks using conventional methods (Fig. 3E). To confirm the functional maturation of the brain organoids, we employed the human synapsin 1 (hSYN1) gene promoter to drive neuronal synapse-specific expression of a red fluorescent protein (RFP) in functional neurons^29^. Consistently, we observed a significant increase in the number of RFP-positive cells in the EMF-exposed AuNP brain organoids (Fig. 3F,G), indicating that electromagnetized AuNPs facilitate efficient functional maturation in 3D cerebral organoids.

### Mechanism of magnetized AuNPs-mediated brain organogenesis

**Figure 4:**
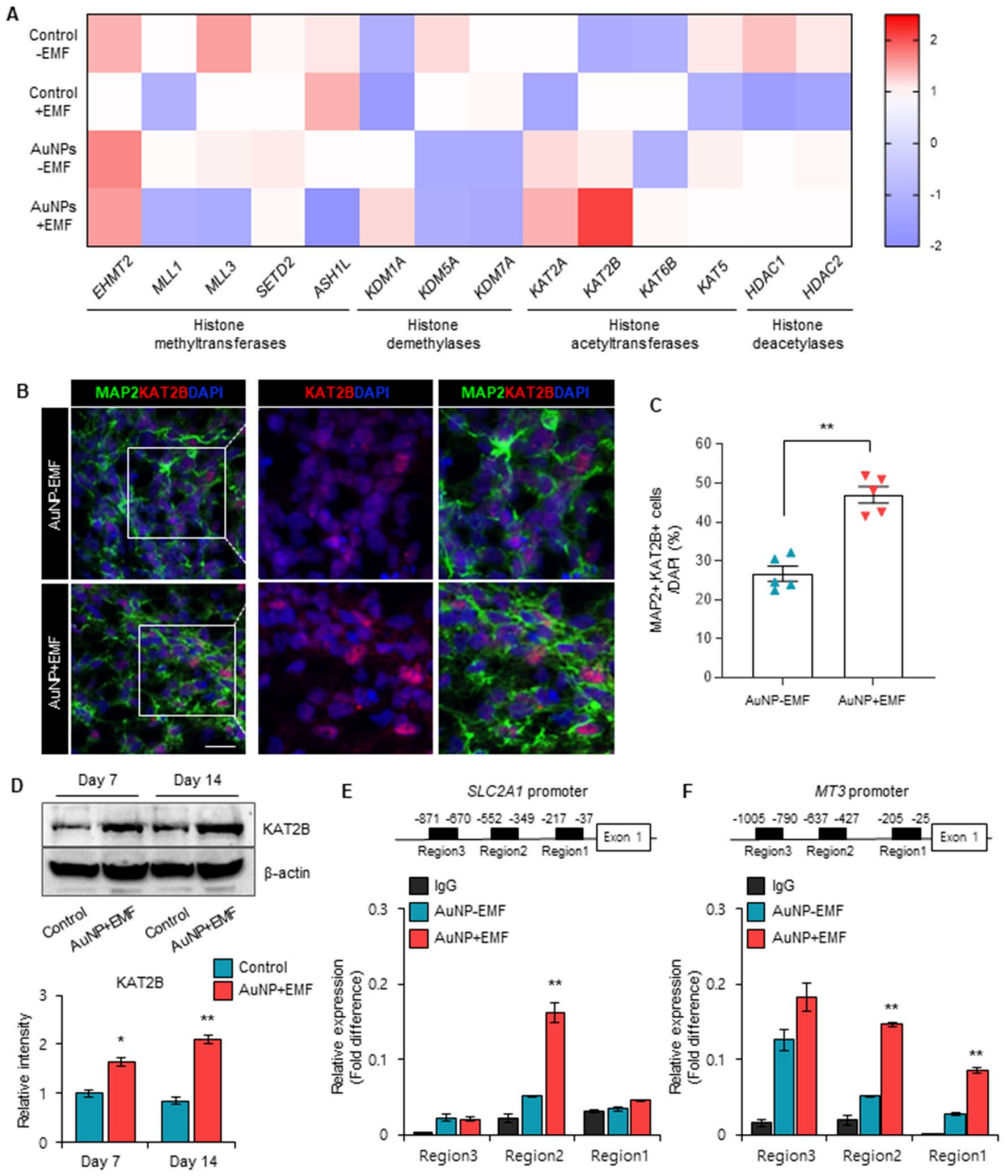
(**A**) Gene expression profiling for histone modifiers, including histone methyltransferases, histone demethylases, histone acetyltransferases, and histone deacetylases. The yellow and dark blue represent higher and lower gene expression levels, respectively. (**B**) Immunofluorescence for MAP2+ and KAT2B + neurons in brain organoids with or without electromagnetized AuNPs. Scale bar = 20 µm. (**C**) Quantification of MAP2 and KAT2B-positive cells in each condition at 3 weeks. Data represent mean ± SEM. Student’s t-test, **P < 0.01; *n* = 5 per group. (**D**) Western blot analysis of KAT2B in brain organoids with or without electromagnetized AuNPs at different time points. Original blots are presented in Supplementary Fig. 4. Data represent mean ± SEM. Student’s t-test, *P < 0.05, **P < 0.01; *n* = 3 per group. (**E**) ChIP-PCR analysis showing the occupancy of KAT2B at the *SLC2A1* promoter regions in brain organoids with or without electromagnetized AuNPs. Data represent mean ± SEM. one-way ANOVA, **P < 0.01; *n* = 3 per group. (**F**) Quantification of the binding of KAT2B at *MT3* promoter regions in brain organoids with or without electromagnetized AuNPs. Data represent mean ± SEM. one-way ANOVA, **P < 0.01; *n* = 3 per group. *Control-EMF* brain organoid without AuNPs and EMF exposure, *Control* + *EMF* EMF-exposed brain organoid without AuNPs-EMF, *AuNP* AuNP-brain organoid without EMF exposure, *AuNP* + *EMF* AuNP-brain organoid with EMF exposure.

To elucidate the mechanism underlying magnetized AuNPs-mediated brain organogenesis, we investigated the potential activation of specific histone modifiers during the development of cerebral organoids. Our analysis revealed a significant upregulation of the lysine acetyltransferase 2B (KAT2B) gene, known for its preferential acetylation of histone H3, in the magnetized AuNP-treated organoids exposed to electromagnetic fields (EMF) (Fig. 4A). However, no noticeable differences were observed in the expression of other epigenetic modifiers (Fig. 4A). In line with these findings, we observed a marked increase in the population of MAP2+ and KAT2B+ cells in the AuNP-treated organoids under EMF exposure conditions (Fig. 4B,C). Furthermore, semiquantitative Western blotting demonstrated higher levels of KMT2B in the EMF-exposed AuNP organoids compared to the control organoids at days 7 and 14 (Fig. 4D). To further investigate the functional implications, we performed chromatin immunoprecipitation (ChIP)-qPCR assays to assess the binding of KAT2B to the promoter regions of neural-specific genes. Interestingly, we found a significant enrichment of KAT2B occupancy at the promoter regions of SLC2A1 and MT3 in the EMF-exposed AuNP organoids (Fig. 4E,F). Collectively, these findings suggest that electromagnetic stimulation facilitated by AuNPs promotes the three-dimensional differentiation of cerebral organoids by modulating the activity of KMT2B and its impact on the accessibility of neural-specific genes. These results imply that histone acetylation may mediate the process of EMF-stimulated AuNP-induced brain organogenesis.

### H3K9 acetylation plays a crucial role in electromagnetized AuNPs-induced brain organogenesis

**Figure 5:**
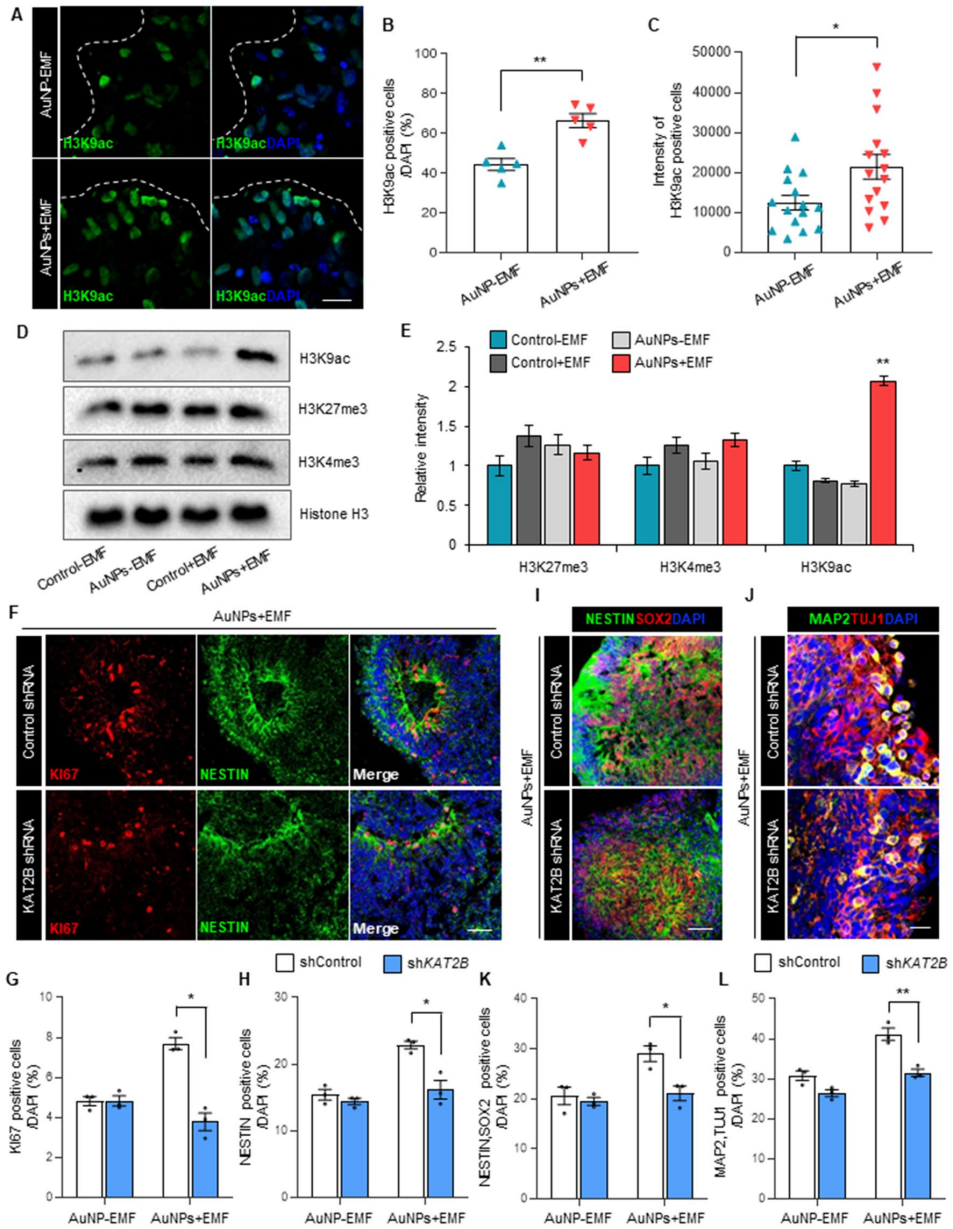
(**A**) Representative images of H3K9ac-positive cells in brain organoids with or without electromagnetized AuNPs. Scale bar = 20 µm. (**B**) Quantification of H3K9ac-positive cells at 3 weeks. Data represent mean ± SEM. Student’s t-test, **P < 0.01; *n* = 5 per group. (**C**) Quantification of the fluorescence intensity of H3K9ac in nuclei using a single confocal section. Data represent mean ± SEM. Student’s t-test, *P < 0.05; *n* = 15 from three samples per group. (**D**) Western blot analysis of histone H3K9 acetylation and histone H3K27 and H3K4 trimethylation in control-EMF, control + EMF, AuNP-EMF, and AuNP + EMF organoids. Original blots are presented in Supplementary Fig. 5. (**E**) The relative intensities of histone H3K9 acetylation and histone H3K27 and H3K4 trimethylation. Data represent mean ± SEM. one-way ANOVA, **P < 0.01; *n* = 3 per group. (**F**) Representative images of KI67− and NESTIN-positive cells in electromagnetized AuNP-organoids treated with *KAT2B*-shRNA. Scale bar = 50 µm. (**G**,**H**) Quantification of KI67 + (**G**) and NESTIN + (**H**) NSCs in AuNP-EMF and AuNP + EMF organoids treated with *KAT2B*-shRNA. Data represent mean ± SEM. two-way ANOVA, *P < 0.05; *n* = 3 per group. (**I**) Representative images of NESTIN− and SOX2-positive cells in electromagnetized AuNP-organoid treated with *KAT2B*-shRNA. Scale bar = 50 µm. (**J**) Representative images of MAP2− and TUJ1-positive cells in electromagnetized AuNP-organoid treated with *KAT2B*-shRNA. Scale bar = 20 µm. (**K**) Quantification of NESTIN+, SOX2 + NSCs in AuNP-EMF and AuNP + EMF organoids treated with *KAT2B*-shRNA. Data represent mean ± SEM. two-way ANOVA, *P < 0.05; *n* = 3 per group. (**L**) Quantification of MAP2+, TUJ1 + neurons in AuNP-EMF and AuNP + EMF organoids treated with *KAT2B*-shRNA. Data represent mean ± SEM. two-way ANOVA, **P < 0.01; *n* = 3 per group. *Control-EMF* brain organoid without AuNPs and EMF exposure, *Control* + *EMF* EMF-exposed brain organoid without AuNPs, *AuNP-EMF* AuNP-brain organoid without EMF exposure, *AuNP* + *EMF* AuNP-brain organoid with EMF exposure.

KAT2B is an acetyltransferase enzyme responsible for H3K9 acetylation (H3K9ac) during neural lineage differentiation^30,31^. To investigate the involvement of H3K9ac in brain organoid development induced by electromagnetized AuNPs, we examined the levels of H3K9ac in cerebral organoids. Remarkably, we found that electromagnetized AuNPs significantly increased the number of H3K9ac-expressing cells in the brain organoids (Fig. 5A,B). Additionally, the relative intensity of H3K9ac-expressing cells was significantly higher in AuNP-treated organoids exposed to EMF (Fig. 5C). This increase in H3K9ac persisted at days 7 and 14 (Fig. S2A), and even at 21 days after exposure to the EMF/AuNP conditions (Fig. 5D,E). Notably, the relative intensities of H3K27me3 and H3K4me3, which are established as key epigenetic markers for repressive and active regulation of neurogenic genes, respectively, did not show significant differences after exposure to electromagnetized AuNPs (Fig. 5E). These findings suggest that EMF stimulation in conjunction with AuNPs contributed specifically to increased histone acetylation in the 3D brain organoids. Furthermore, we examined the expression of oncogenes, DNA damage marker, and inflammatory marker such as Brca1, Parp1, and Cox2 in magnetized 3D brain organoids^32,33^. However, we did not observe significant differences in the relative expression of these genes following exposure to electromagnetized AuNPs (Fig. S2B).

To further investigate the functional role of KAT2B in electromagnetized AuNP-treated brain organoids under EMF stimulation, we employed a shRNA lentivirus to knockdown KAT2B expression. Western blot analysis confirmed the differential expression of KAT2B upon knockdown (Fig. S3A,B). Notably, the observed increase in H3K9ac levels under electromagnetized AuNP conditions induced by EMF was significantly diminished in the 3D organoids following KAT2B knockdown (Fig. S3C). Moreover, we observed a significant reduction in the population of NESTIN- and KI67-positive cells in the electromagnetized AuNP organoids upon KAT2B knockdown (Fig. 5F–H). Consistently, the numbers of NESTIN+ and SOX2+ cells were significantly decreased in the electromagnetized AuNP organoids treated with KAT2B shRNA (Fig. 5I,K). Similarly, inhibition of KAT2B led to a significant decrease in the number of MAP2− and TUJ1-positive neurons in the electromagnetized AuNP organoids (Fig. 5J,L). These results suggest that electromagnetized AuNPs functionally activate the development of 3D organoids through the involvement of KAT2B, which plays a critical role in the regulation of neural-specific loci under EMF exposure conditions.

## Conclusions

Brain organoids offer a valuable opportunity to model the unique characteristics of brain development and diseases. However, existing protocols for generating brain organoids have limitations, including slow maturation and inconsistent results. In this study, we present a significant advancement by demonstrating that EL-EMF mediated magnetized AuNPs greatly enhance the efficiency of neuronal differentiation and maturation in cerebral organoids compared to conventional methods. Extremely low-frequency electromagnetic fields (EL-EMF) have distinct advantages over static fields or other magnetic treatments when it comes to influencing cellular responses. Firstly, EL-EMF induces dynamic changes in the electromagnetic environment, providing a versatile and nuanced modulation of cellular responses compared to static fields. Secondly, EL-EMF can penetrate tissues more effectively, allowing for deeper interaction with cells and tissues, making it suitable for studying complex cellular systems and three-dimensional tissue constructs^34^. Additionally, EL-EMF interacts with cellular components and biomolecules, triggering intracellular signaling pathways and molecular events that lead to diverse cellular responses^35^. In contrast, static fields or other magnetic treatments may lack the specificity and effectiveness in modulating these complex cellular processes^36,37^. These unique characteristics of EL-EMF make it a crucial tool for investigating and manipulating cellular responses in various biological contexts.

Furthermore, we have discovered the involvement of a histone modifier, KAT2B, which responds to the stimulation of AuNPs under EMF exposure. This finding suggests that effective generation of mature brain-like structures is achieved through epigenetic histone acetylation. However, the precise mechanisms by which electromagnetic fields (EMF) influence neuronal migration and proliferation are not fully understood. Nonetheless, it is clear that EMF-induced KAT2B-mediated histone acetylation directly influences neuronal proliferation and migration. Our experimental results consistently demonstrate the binding of KAT2B to the promoter regions of neural-specific genes during EMF-induced 3D brain organogenesis. These genes play a crucial role in promoting neural development, resulting in an enlargement of the organoid diameter and an upregulation of neural stem cell gene expression in response to EMF treatment. Taken together, our findings provide a powerful platform to investigate the mechanisms underlying brain development and diseases, as well as to validate robust tools for developing medicines.

## Materials and methods

### AuNP synthesis and preparation of RGD-AuNPs

Following the previous methods, the CYGRGDS peptide was synthesized according to the standard 9-fluorenylmethoxycarbonyl-based solid phase peptide synthesis protocol, on a Rink amide AM resin (0.1 mmol/g). In addition, 20 nm AuNPs were also prepared following a standard protocol^38,39^. Briefly, an aqueous solution of 0.3 mM HAuCl_4_·3H_2_O was brought to a boil with vigorous stirring, and a 10 mM sodium citrate solution was added. After boiling for 7 min, the heating resource was removed, and the colloid was stirred for another 15 min. The AuNP solution was characterized by UV–Vis spectroscopy and an absorbance maximum at 520 nm were obtained. The size and surface charge of the AuNPs was measured using dynamic light scattering. Arginine-glycine-aspartate (RGD)-conjugated AuNPs were obtained from citrate-coated AuNPs through a modified place exchange reaction with thiol ligands^40^. Briefly, mPEG_350_-SH solution (10 mM, 0.1 mL) was added to the citrate-coated AuNP solution (28.3 μg/mL, 2 mL) and gently mixed. After incubating at 4 °C for 24 h, the supernatant was removed by centrifugation and the RGD peptide (0.5 mM, 1 mL) was mixed with the PEG-coated AuNPs. After 24 h treatment, the modified AuNPs were washed with ddH_2_O and resuspended in ddH_2_O. Arginine-glycine-aspartate (RGD)-conjugated AuNPs were characterized by UV–Vis spectroscopy, dynamic light scattering, and ζ-potential measurement.

### Lentivirus generation and viral infection

*KAT2B* shRNA (Target sequence: GCAGATACCAAACAAGTTTAT) was purchased from Applied Biological Materials Inc. Lentivirus production was prepared as described previously^41^. Briefly, the lentivirus was produced in HEK293T cells grown in Dulbecco’s Modified Eagle Medium containing 10% fetal bovine serum and 1% penicillin/streptomycin. Afterward, the cells were transfected with the lentivirus construct, *KAT2B* shRNA, and packaging plasmid, psPAX2, and pMD2.G vectors using calcium phosphate co-precipitation. One day after transfection, the medium was changed, and the viruses were harvested 48–72 h later. After approximately 24 h EMF exposure, the embedded AuNP brain organoids with Matrigel were infected with the lentivirus, *KAT2B* shRNA, twice in 2 days.

### Generation of electromagnetized AuNP-human cerebral organoids

Human control fibroblasts (GM23967, Male, 52YR) were purchased from the Coriell Cell Repository and cultured in human cell culture medium containing Dulbecco’s Modified Eagle Medium, 10% fetal bovine serum, 1% nonessential amino acids (Gibco), 0.1% β-mercaptoethanol (Gibco), and 1% penicillin/streptomycin (Gibco). To generate hiPSCs from fibroblasts, the cells were infected with a lentivirus construct harboring Oct4, Sox2, C-myc, and Klf4, twice over 2 days. The hiPSC lines were cultured in StemMACS iPS-Brew XF (Miltenyi Biotec, 130-104-368), containing StemMACS Supplement (Miltenyi Biotec) on Matrigel Matrix (Corning, #354234) coated plates at 37 °C and 5% CO_2_. The hiPSCs were singularized using Accutase (Stemcell Technologies, #07920) to induce their dissociation into single cells, while the EBs were maintained in a human embryonic stem cell growth medium without a ROCK inhibitor for 3 days. A single embryoid body (EB) measuring 450–500 µm in diameter was transferred to pre-warmed 6-well tissue culture plates containing 5 µL of Human ESC medium supplemented with 1 µL of RGD-AuNPs nanoparticles (at a concentration of 8 × 10^12^ nanoparticles/mL). To prepared electromagnetized AuNP-human cerebral organoids, the EB-AuNPs mixture was embedded in a 10 µL Matrigel matrix and allowed to polymerize for 10 min at room temperature. The EBs conjugated with the RGD-AuNPs were maintained in an incubator at 37 °C and 5% CO_2_. Human embryonic stem cell growth medium was replaced with neural stem cell medium (Advanced DMEM/F12 and neurobasal medium, 1 × N2, 1 × B27, 5% Albumax-I, 2 mM GlutaMAX, 0.1 mM beta-mercaptoethanol, 3 µM CHIR99021, 0.5 µM A83-01, and 10 ng/mL LIF) containing fibroblast growth factor-2 (20 ng/mL) and epidermal growth factor (20 ng/mL) for 5 days. To promote organoid patterning, aggregated cells were cultured in a neural stem cell medium containing 200 nM ascorbic acid and 20 ng/mL brain-derived neurotrophic factor for 1 week. To promote organoid maturation, organoids were cultured in cerebral organoid differentiation medium (Advanced DMEM/F12 and neurobasal medium, 1 × N2, 1 × B27, 5% Albumax-I, 2 mM GlutaMAX, 0.1 mM beta-mercaptoethanol) containing 200 nM ascorbic acid, 20 ng/mL brain-derived neurotrophic factor, and 20 ng/mL glial cell-derived neurotrophic factor from day 12 to day 21. The culture medium was changed every three days, and the organoids were maintained on an orbital shaker (PSU-10i, Biosan) in an incubator at 37 °C and 5% CO_2_.

### Flow cytometry

The organoids were dissociated using papain for 15 min, with pipetting every 5 min to facilitate cell dissociation. Dissociated organoids were washed with 1 × DPBS (Gibco, 14190-144), then, filtered using a cell strainer (SPL, 352340) to remove cell debris. The remaining cells were resuspended in 4% paraformaldehyde in phosphate-buffered saline and incubated for 10 min. The fixed cells were washed twice with 1% bovine serum albumin in phosphate-buffered saline and resuspended in fluorescence-activated cell sorting buffer. Flow cytometry was performed using Accuri equipment (Becton–Dickinson), and data analysis was conducted by FlowJo vX software (TreeStar).

### Immunofluorescent staining analysis

Organoids were fixed in 4% paraformaldehyde for 1 h after being washed with 1 × phosphate-buffered saline and then incubated in a 30% sucrose solution overnight. Following the previously published protocol^13^, organoid sections were immunostained using the following primary antibodies: PAX6 (Invitrogen, 42-6600), NESTIN (Invitrogen, PA5-47378), KI67 (Invitrogen, 14-5698-82), SOX2 (Millipore, AB5603), TUJ1 (Sigma, T2200), MAP2 (Cell Signaling, 4542 s), DCX (Cell signaling, 4604), VGLUT1 (Invitrogen, 48-2400), TBR1 (Abcam, ab31940), NEUN (Millipore, MAP377 KAT2B (Abcam, ab12188), and H3K9ac (Abcam, ab4441). Appropriate secondary antibodies (Invitrogen) were used for amplifying the signal. Next, nuclei were labeled with 6-diamidino-2-phenylindole (DAPI, Invitrogen). Sections were mounted in Fluoromount-G mounting medium and images were captured using a confocal laser scanning microscope (ZEISS, LSM800). The number of Nesin positive cells were counted in three, non-overlapping visual field at a magnification of × 20. The percentages of immunopositive cells were calculated per 100 cells, visualized with DAPI staining^42^.

### Western blot analysis

Organoid samples for all conditions were lysed in lysis buffer (1% NP-40, 0.5% DOC, 0.1% SDS, 150 mmol/L NaCl in 50 mmol/L Tris, pH 8.0, Sigma-Aldrich; and 1 × proteinase inhibitor mixture, Roche) after being dissociated with a homogenizer and washed with 1 × phosphate-buffered saline. After adding 5 × SDS loading buffer and boiling at 99 °C for 5 min, the extracted proteins were electrophoresed on a 12% sodium dodecyl sulfate–polyacrylamide gel and transferred to nitrocellulose membranes (GE Healthcare Bio-Sciences). The membranes were probed with the following primary antibodies: anti-KAT2B (1:500, Abcam, ab12188), anti-H3K9ac (1:1000, Abcam, ab4441), anti-H3K27me3 (1:1000, Millipore, 07-449), anti-H3K4me3 (1:500, Abcam, Ab8580), anti-histone H3 (1:1000, AbFrontier, LF-MA02330), and beta-actin (1:1000, AbFrontier, LF-PA0207).

### Quantitative real-time polymerase chain reaction analysis

A previously published protocol was used to isolate total RNA from organoid samples^43^. To synthesize complementary DNA from isolated RNA, the AccuPower RT-PCR PreMix (Bioneer) was used according to the manufacturer’s protocols. Quantitative RT-PCR was performed using Platinum SYBR green qPCR SuperMix (Invitrogen) in a Rotor-Gene Q real-time PCR cycler (QIAGEN). Expression of all target genes was normalized against the expression of glyceraldehyde-3-phosphate dehydrogenase, GAPDH, in each organoid sample.

### Statistical analysis

All data are presented as the mean ± SEM of each independent experiment. Values of ‘n’ indicate the number of independent experiments performed or the number of individual experiments. Statistical analyses were performed with GraphPad Prism. The p-values were calculated by Student’s t-test, one-way ANOVA, and two-way ANOVA tests followed by Tukey–Kramer multiple comparisons test. All of the statistical details for each experiment have been documented in the individual figure legends.

### Supplementary Information

Supplementary Figures.

## Data Availability

The authors have no data to share since all data are shown in the submitted manuscript.
