# Supplementary material for: Efficient generation of brain organoids using magnetized gold nanoparticles
Source: Sci Rep. 2023 Dec 1;13:21240. doi: 10.1038/s41598-023-48655-8 (PMC10692130; doi:10.1038/s41598-023-48655-8)
Supplement: Supplementary file 1 — Supplementary Figures. [file 41598_2023_48655_MOESM1_ESM.docx]

**Efficient generation of brain organoids using magnetized gold nanoparticles**

Hongwon Kim^1,2^, Yoo-Jung Lee^1^, Youngeun Kwon^3^, and Jongpil Kim^1*^

**Supplementary Information.**

**
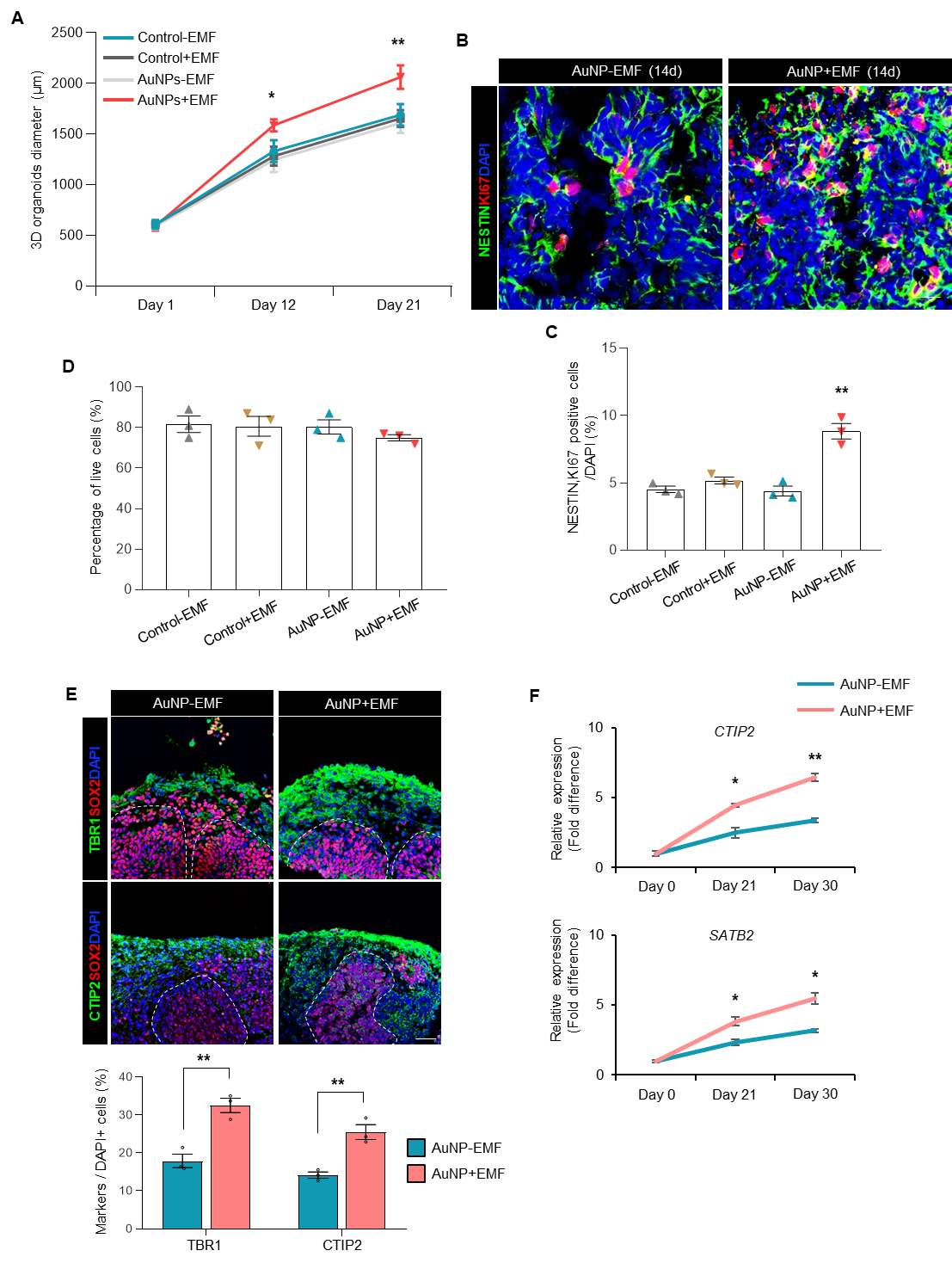
**

**Supplementary figure 1.**

(A) Measurement of 3D organoid diameter at different time points. Data represent mean ± SEM. one-way ANOVA, **P* < 0.05, ***P* < 0.01; *n* = 5 per group. (B) Immunofluorescence for KI67 and NESTIN in the control and electromagnetized AuNP organoids at 2 weeks. Scale bar = 20 µm. (C) Quantifications of the KI67+, NESTIN+ cells in control organoids and electromagnetized AuNP organoids. Data represent mean ± SEM. one-way ANOVA, ***P* < 0.01; *n* = 3 per group. (D) Graph showing live/dead analysis resulting from the control and electromagnetized AuNP organoids at 2 weeks. Data represent mean ± SEM. one-way ANOVA. *n* = 3 per group. (E) Immunofluorescence for cortical layer markers, TBR1 and CTIP2, in the control and electromagnetized AuNP organoids. Scale bar = 50 µm. (F) qRT-PCR analysis of the cortical layer markers, *CTIP2 and SATB2*, at different time points. Data represent mean ± SEM. Student’s t-test, **P* < 0.05, ***P* < 0.01; *n* = 3 per group.

**
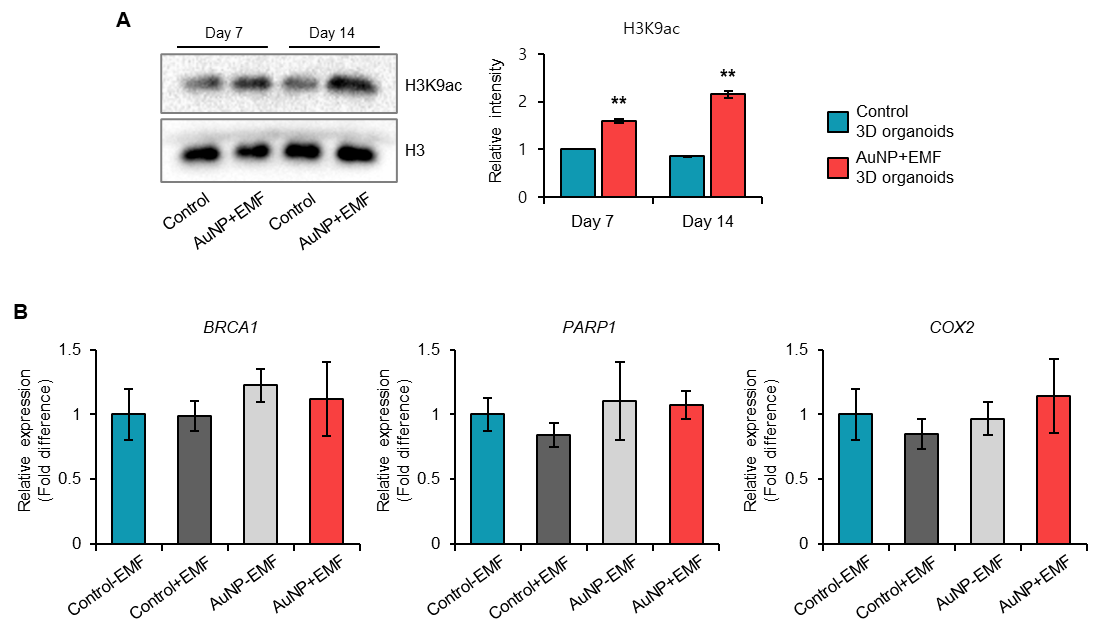
**

**Supplementary figure 2.**

(A) Western blot analysis of histone H3K9 acetylation in brain organoids with or without electromagnetized AuNPs at day 7 and day 14. Original blots are presented in Supplementary Figure 6. Data represent mean ± SEM. Student’s t-test, ***P* < 0.01; *n* = 3 per group. (B) qRT-PCR analysis of DNA damage response markers, BRCA1 and PARP1, and inflammatory marker, COX2, in control-EMF, control + EMF, AuNP-EMF, and AuNP + EMF organoids. Data represent mean ± SEM. one-way ANOVA. *n* = 3 per group.

**
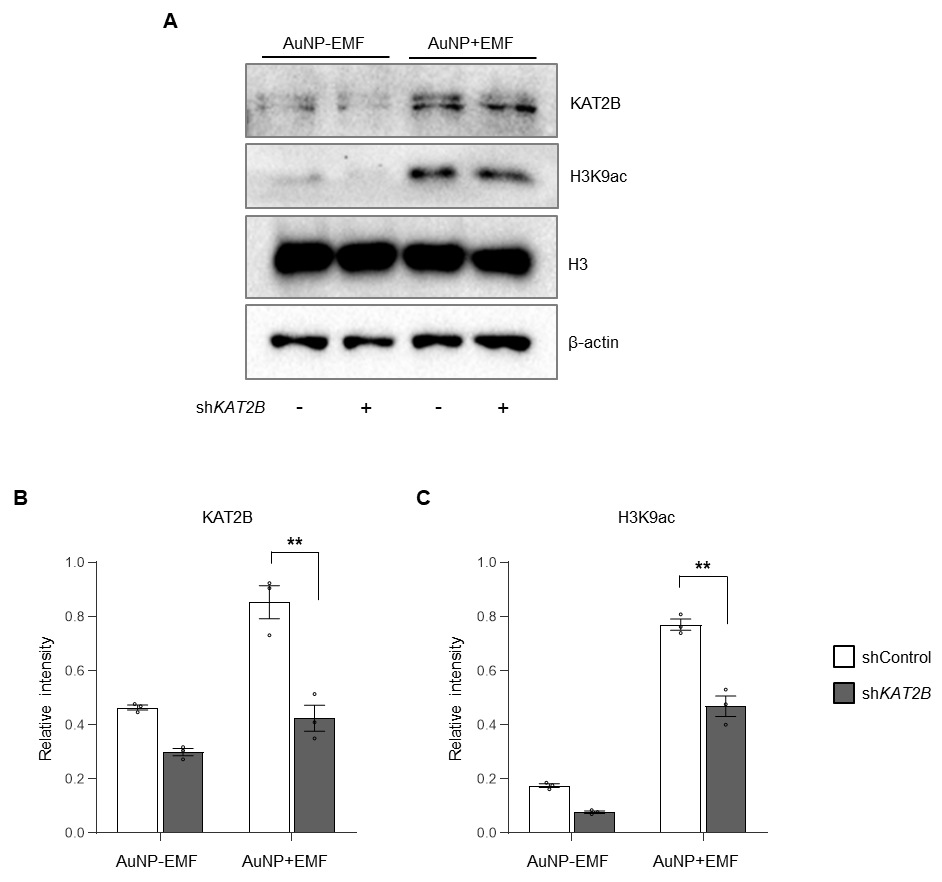
**

**Supplementary figure 3.**

(A) Western blot analysis of KAT2B and histone H3K9 acetylation in AuNP-EMF and AuNP + EMF organoids treated with *KAT2B*-shRNA. Original blots are presented in Supplementary Figure 7. (B and C) The relative intensities of KAT2B and histone H3K9 acetylation in AuNP-EMF and AuNP + EMF organoids treated with *KAT2B*-shRNA. Data represent mean ± SEM. ANOVA-test, ***P* < 0.01; *n* = 3 per group.


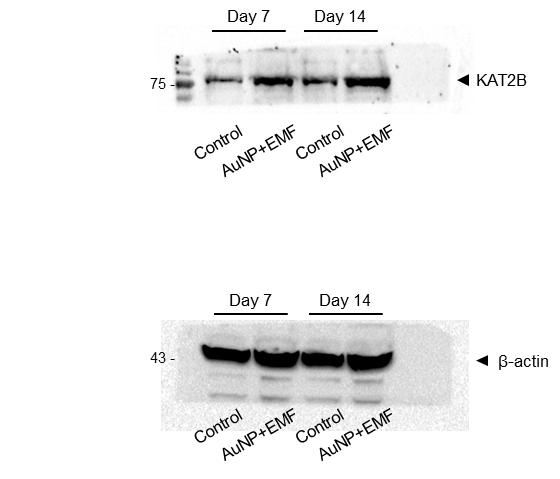


**Supplementary figure 4.** The expression levels of KAT2B were detected by Western blot analysis in brain organoids with or without electromagnetized AuNPs at different time points, β-actin used as the reference gene.

**
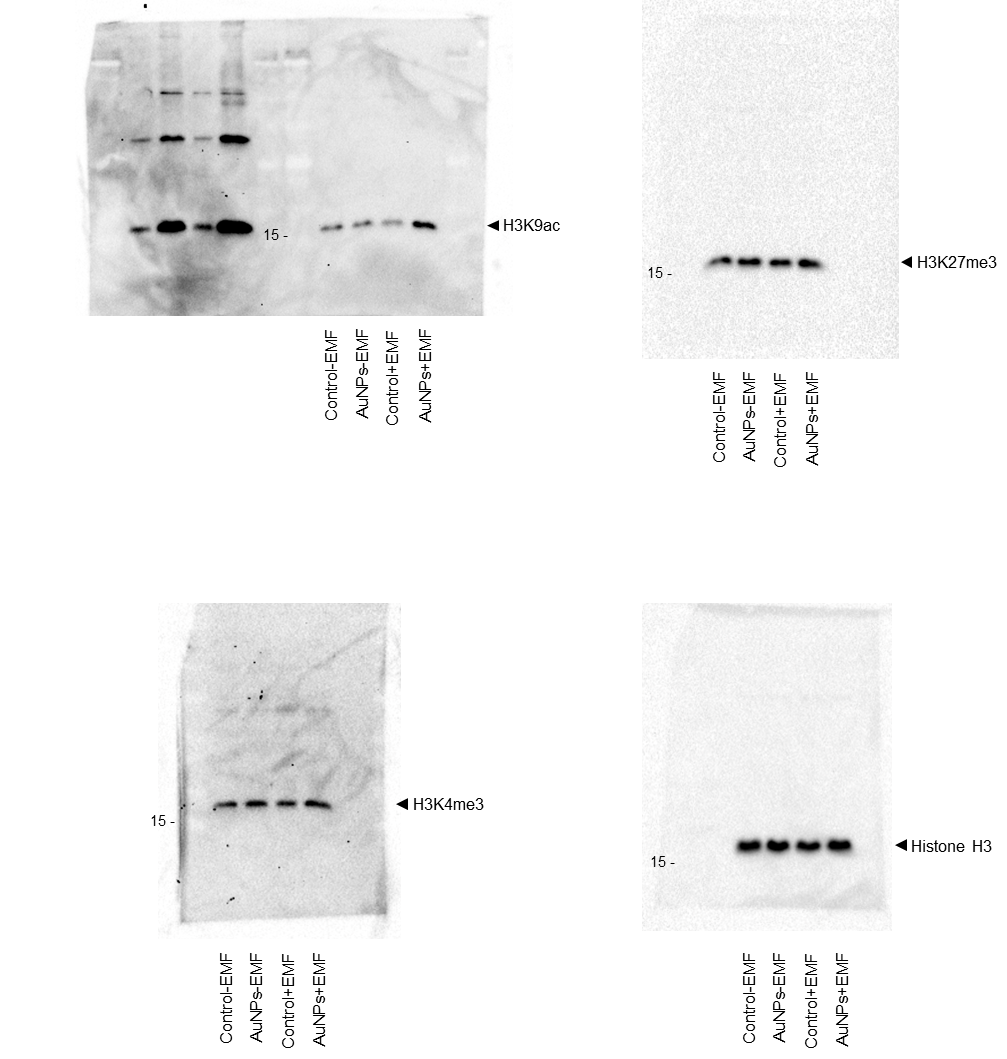
**

**Supplementary figure 5.** The expression levels of H3K9ac, H3K27me3, H3K4me3 were detected by Western blot analysis, Histone H3 used as the reference gene.

**
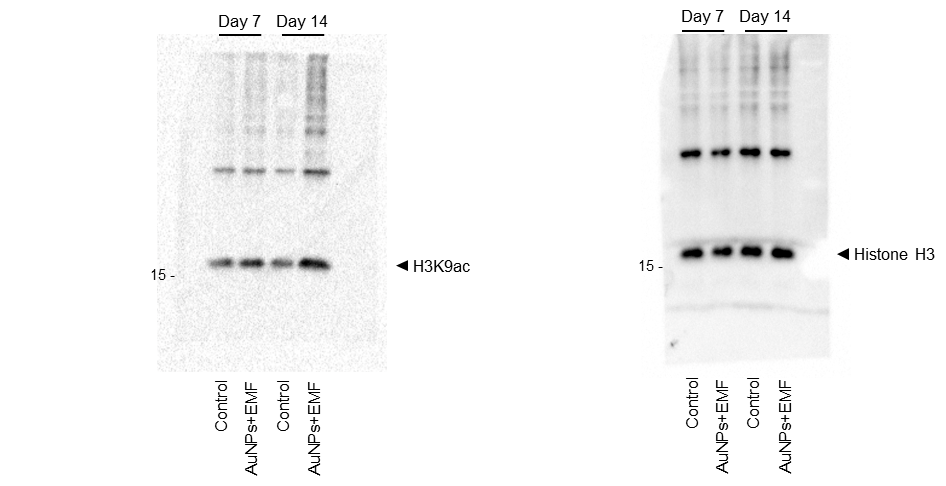
**

**Supplementary figure 6.** The expression levels of H3K9ac were detected by Western blot analysis in brain organoids with or without electromagnetized AuNPs at different time points, Histone H3 used as the reference gene.

**
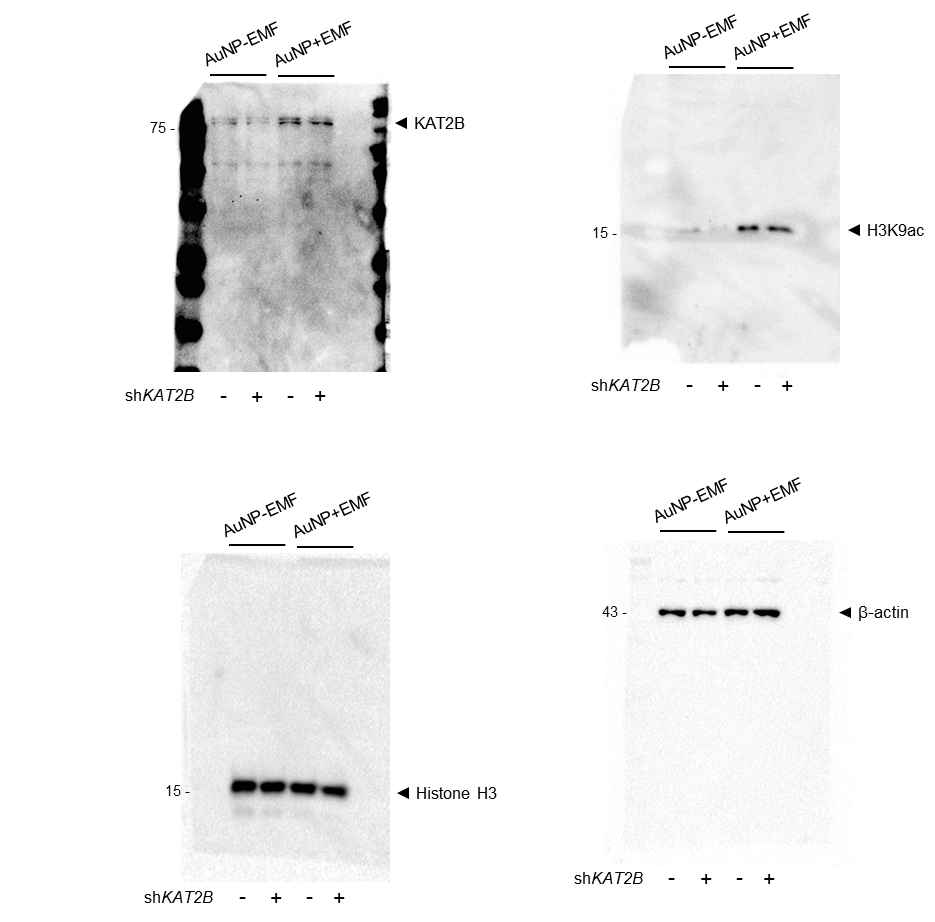
**

**Supplementary figure 7.** Western blot analysis of KAT2B and histone H3K9 acetylation in AuNP-EMF and AuNP + EMF organoids treated with *KAT2B*-shRNA.
